# Immune consequences of CT-guided radiation therapy of mouse mammary tumors

**DOI:** 10.1186/2051-1426-1-S1-P116

**Published:** 2013-11-07

**Authors:** Michael J Gough, Kristina H Young, Benjamin L Cottam, Talicia Savage, Jason R Baird, David Friedman, Marka R Crittenden

**Affiliations:** 1 Earle A Chiles Research Institute, Providence Cancer Center, Portland, OR, USA; 2 The Oregon Clinic, Portland, OR, USA; 3 Department of Radiation Medicine, Oregon Health and Sciences University, Portland, OR, USA

## 

Transgenic tumor models provide the closest animal approximation of human cancer development and progression, and a challenging treatment model. The orthotopic location of these tumors presents challenges in testing radiation therapies because conventional radiation models using patient linear accelerators or untargeted units with selective shielding are difficult or impractical in transgenic tumor models. We have tested CT-guided radiation therapy using a Small Animal Radiation Research Platform to treat transgenic MMTV-PyMT mammary tumors. Image guiding permits close targeting of tumors with no measurable toxicities at single tumor doses tested up to 20Gy. We demonstrate that tumor treatment results in a dose-dependent control of mammary tumors. Histology illustrates destruction of invasive carcinoma in the mammary gland with remaining tissue features of premalignant disease due to ongoing transgene-driven tumorigenic progression in other mammary cells. We demonstrate progressive expansion of CD11b+Gr1+ myeloid cells detectable in the blood of mice that correlates with tumor progression, and that focal treatment of mammary tumors with radiation therapy reverses this myeloid expansion without affecting systemic T cell numbers. The result is an improved systemic myeloid cell: T cell ratio in treated mice, which has been associated with improved immune function in many animal models of cancer. This platform provides an approach to study hypofractionated radiation therapy in authentic animal models of tumor progression without causing systemic lymphopenia and permits experiments investigating the interaction between radiation therapy and endogenous immune responses in transgenic and orthotopic tumor models.

